# Epigenetic Mechanisms Link Maternal Diets and Gut Microbiome to Obesity in the Offspring

**DOI:** 10.3389/fgene.2018.00342

**Published:** 2018-08-27

**Authors:** Yuanyuan Li

**Affiliations:** ^1^Department of Pharmacology and Toxicology, University of Alabama at Birmingham, Birmingham, AL, United States; ^2^Comprehensive Cancer Center, University of Alabama at Birmingham, Birmingham, AL, United States; ^3^Nutrition Obesity Research Center, University of Alabama at Birmingham, Birmingham, AL, United States

**Keywords:** nutrition, maternal, microbiome, epigenetics, obesity

## Abstract

Nutrition is the most important environmental factor that can influence early developmental processes through regulation of epigenetic mechanisms during pregnancy and neonatal periods. Maternal diets or nutritional compositions contribute to the establishment of the epigenetic profiles in the fetus that have a profound impact on individual susceptibility to certain diseases or disorders in the offspring later in life. Obesity is considered a global epidemic that impairs human life quality and also increases risk of development of many human diseases such as diabetes and cardiovascular diseases. Studies have shown that maternal nutrition status is closely associated with obesity in progenies indicating obesity has a developmental origin. Maternal diets may also impact the early establishment of the fetal and neonatal microbiome leading to specific epigenetic signatures that may potentially predispose to the development of late-life obesity. This article will review the association of different maternal dietary statuses including essential nutritional quantity and specific dietary components with gut microbiome in determining epigenetic impacts on offspring susceptibility to obesity.

## Introduction

Mammalian embryonic development and subsequent fetal development involve complicated molecular regulations that occur through precise controlled sequential events including fertilization, implantation, gastrulation, and organogenesis leading to a complete developmental process of a new life in mammals. Interactions between intrinsic factors such as genetics and epigenetics and extrinsic maternal factors such as maternal nutrition greatly influence these developmental processes, which may culminate in abnormalities or disadvantages during *in utero* development leading to increased risks for certain diseases or disorders in later-life (Bateson et al., 2004; Li et al., 2014; Indrio et al., 2017). This “Developmental Origins” hypothesis based on the interaction between early developmental plasticity, the environmental factors and the outcome of health and disease has been widely accepted over the last decades (Bateson et al., 2004; Barker, 2007).

Importantly, maternal factors such as maternal nutrition status have been increasingly implicated in influencing offspring susceptibility to certain diseases or disorders. The study on maternal nutrition and obesity development is one of the most important areas highlighting the impact of maternal nutritional factors on the early transmission of obesity (Parlee and MacDougald, 2014). Obesity is a complex metabolic disorder, and is closely linked to the most common human diseases such as diabetes, cardiovascular diseases and cancer (Astrup and Finer, 2000; Apovian, 2013). Studies have shown that obesity has strong inherited tendency and the development of obesity in adult life can be influenced by fetal (*in utero*) exposure to environmental factors such as maternal diets (Mühlhäusler et al., 2008; Parlee and MacDougald, 2014). In addition, the effects of maternal nutrition depend on its qualitative factors such as certain bioactive diets with epigenetic regulatory properties as well as its quantitative factors such as under- and over-nutrition aspects, eventually influencing the development of obesity in the progeny’s later life (Parlee and MacDougald, 2014).

It is well demonstrated that nutrition as one of the most important environmental factors influences adult-onset disease. Because the genome is evolutionarily and chemically stable, nutritional factors do not generally cause genomic changes in DNA sequences but rather cause changes via an independent manipulation of DNA sequence such as epigenetics (Skinner et al., 2010). Epigenetics defines a variety of processes that lead to heritable changes in gene expression without changing DNA sequencing (Egger et al., 2004). DNA methylation, histone modification and non-coding RNA are the main mechanisms for epigenetic modifications. A large body of evidence has demonstrated the importance of nutritional factors on regulation of epigenetic mechanisms leading to various beneficial effects such as disease prevention (Jirtle and Skinner, 2007; Li and Tollefsbol, 2010). Most importantly, since epigenetic events frequently occur during early developmental stages (Li, 2002; Santos et al., 2002), early nutritional exposure during this crucial time period may alter epigenetic modification patterns during developmental programming (Li et al., 2014; Lee, 2015), thereby potentially causing differential individual susceptibility to the later development of human disease, such as obesity and obesity-associated metabolic diseases.

Recent studies have clearly shown that the gut microbiota can greatly affect host metabolism via microbiome metabolites (Bäckhed, 2011). A dysbiotic microbiota plays a central role in the development of obesity (Kvit and Kharchenko, 2017). A growing body of evidence indicates that early environmental factors such as maternal diets can influence gut microbiota composition in the offspring through maternal gut microbiota transfer and, in turn, these changes can persist until adulthood and exert long-term effects on metabolic health and disease development (Chu et al., 2016b; Zhou and Xiao, 2018).

Although the possible mechanisms through which intestinal bacteria influence human health are still under investigation, epigenetic mechanisms prevail and have drawn extensive attention recently. The potential mechanisms for gut microbes-induced epigenetic changes in the host have been recently linked to specific pattern changes of epigenetic modifications after exposure to certain bacteria through direct or indirect mechanisms, suggesting the existence of complex interactions between the microbiome, metabolism and epigenome (Cortese et al., 2016).

It raises the hypothesis that improving maternal metabolism through maternal diets and gut microbiome may benefit the burden of disease. Thus, exploration of the epigenetic mechanisms through which beneficial outcomes such as obesity prevention in offspring are reprogrammed and possibly persistent in future generations will have important translational potential. More importantly, exposure to bioactive diets with potential epigenetic modulatory properties, the so called “epigenetic diets” (Li and Tollefsbol, 2010; Hardy and Tollefsbol, 2011), during early developmental stages may affect key gene expression through interaction with the epigenome, microbiome and metabolome network leading to phenotypic changes in the offspring such as a reduced risk in obesity. Thus, elucidating the correlation between maternal diets, gut microbiome and epigenetics may provide valuable insight into the mechanisms involved in the propagation of obesity across generations.

This review aims to provide an overview of the complicated network between maternal nutrition, microbiome and epigenome, which may shed light on the important roles of early-life epigenetic control by appropriate administration of prenatal and/or postnatal maternal diets leading to early obesity prevention in the offspring.

## Early-Life Nutrition and Obesity

Throughout the human lifespan, nutrition is one of the most important environmental factors that influences adult-onset disease outcome. Nevertheless, the effects of nutritional exposures during prenatal and early postnatal periods are most profound resulting in long-term phenotypic changes in the offspring. It has become increasingly apparent that maternal intrauterine stimuli will affect the fetus’s response to future predictive factors resulting in permanent changes in fetus’s genome and epigenome that may improve the adaptation to the postnatal environment (Li et al., 2014; Parlee and MacDougald, 2014; Lee, 2015; Indrio et al., 2017). These adjustments could lead to either advantageous or disadvantageous outcomes that influence heritable risk factors for future diseases such as obesity. Thus, a feasible maternal diet regimen in prevention of later-life obesity is urgently needed.

## Origin of Obesity

Obesity has become a worldwide epidemic that is characterized by excess white adipose tissue and a result of chronic disease and energy imbalance (Stevens et al., 2012). As a major risk factor for metabolic syndrome, obesity is associated with various metabolic disorders including insulin resistance, hyperglycemia, hyperlipidemia, and hypertension as well as many chronic diseases such as diabetes, cardiovascular disease, and cancers (Astrup and Finer, 2000; Apovian, 2013).

It is commonly accepted that obesity is the result of interaction among multiple factors including genetic, environmental, behavioral, physiological and social-economic factors that lead to energy imbalance and promote excessive fat deposition. However, the origin of development of obesity can retrospect to the early developmental stages in the human lifespan (Jirtle and Skinner, 2007). Barker and his colleagues proposed the concept of “the thrifty phenotype hypothesis” indicating that maternal nutrition imbalance during pregnancy and early postpartum may cause mal-adaption of phenotypic plasticity to this thrifty environment leading to disease and dysfunction in later life (Barker, 2004, 2007). It is believed that individual early embryonic and fetal environmental exposure greatly affects metabolic thriftiness causing genetic/epigenetic reprogramming during early development. The adverse impact may extend to later life and influence an adult nutrient status leading to disregulation of metabolic systems and obesity outcome. Thus, interactions between early-life nutrition and genetic/epigenetic alterations in response to the fluctuating environmental factors during early development eventually determine adipose tissue development, central control of energy balance and obesity in adult life.

## Epigenetic Reprogramming During Early Developmental Stage

Epigenetics define the heritage changes in gene expression that are independent from changes in DNA sequencing such as mutation. Different from genetics, epigenetic alterations regulate gene expression and change phenotypes without changes in genotypes, which provide an additional layer of gene regulation mechanisms in addition to those of genetics.

Epigenetic reprogramming during early development is the best characterized epigenetic function that influences early gene expression patterns leading to a broad developmental potential in the organisms (Li, 2002; Santos et al., 2002). Even before development, the parental epigenomic profiles will be re-written in the matured gametes by erasing of somatic epigenetic signatures in the primordial germ cells (PGCs) via a comprehensive demethylation process, and reestablishment of sex- and germ cell-specific epigenetic signatures for preparation of sexual reproduction via a remethylation process. After fertilization, the embryo will undergo a genome-wide DNA methylation reprogramming process followed by removal of almost all existing parental epigenetic signatures except for the imprinting markers and reestablishment of a new methylation profile to trigger the embryonic developmental program. During the progression of embryonic development, a totipotent-state embryo mass will be differentiated toward the future lineages directed by the reprogrammed DNA methylation profile which provides a fundamental epigenetic barrier that guides differentiation and also prevents regression into an undifferentiated state. The established epigenetic landmarks are relatively stable and heritable through mitosis, allowing a faithful differentiation process and the propagation of lineage-specific transcription profiles over many cell divisions during the whole lifetime in the organisms.

**FIGURE 1:**
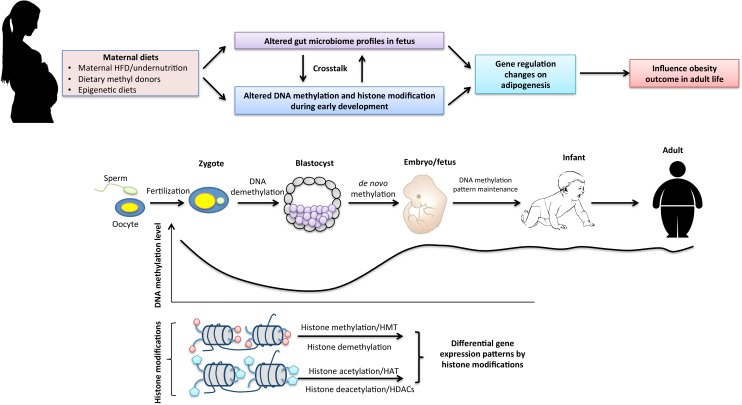
The interaction of maternal dietary factors with early-life epigenetic mechanism and the gut microbiome in regulation of obesity in adult life. Epigenetics undergo dramatic changes during early embryogenesis. Genomic DNA undergoes global demethylation and subsequent *de novo* remethylation processes to maintain basal DNA methylation patterns throughout the life span. Histone modifications that maintain transcriptional regulations of cell-differentiation lineages during early development are mainly regulated by histone methylation and acetylation. Maternal nutritional status may affect early epigenetic reprogramming processes as well as early establishment of the gut microbiome in the fetus that result in gene expression changes on adipogenesis and metabolisms leading to altered phenotypes such as different susceptibility to obesity and obesity-related metabolic disease in adult life.

Thus, the most critical time for reestablishment of genome-wide epigenetic profiles occurs during early embryogenesis. A single mismatch during this time may result in disadvantageous changes including embryonic lethality (Li et al., 1992), developmental malformations (Matsuda and Yasutomi, 1992) and increased risk for certain diseases (Gopalakrishnan et al., 2008). To the contrary, a beneficial rewriting in the early-life epigenome may lead to advantageous outcomes such as disease prevention (Li et al., 2014). Since epigenetic mechanisms are frequently regulated by environmental factors, maternal diets and early-life nutrition intervention may play an important role in influencing early-life epigenetic reprogramming processes leading to different phenotypic changes such as varied disease risk in the offspring (**Figure 1**).

## Maternal Diet, Obesity and Epigenetics

### Maternal Over-Nutrition

With changes in diet and lifestyle, global nutrient excess and obesity are emerging problems that threaten human health. Maternal obesity is commonly seen in the United States and is considered as a strong predictor for a poor intrauterine environment and adverse outcomes for both the mother and child (Hussen et al., 2015).

Numerous human studies have shown that maternal obesity likely leads to detrimental pregnancy outcomes such as increased risks of gestational diabetes, gestational hypertension, preeclampsia and cesarean section compared to pregnancy with normal weight (Gaillard et al., 2013). In addition, excess maternal nutrition is a predisposing factor for progeny to develop high birth weight and obesity in later life (Laraia et al., 2007), as well as the other severe adverse consequences, such as preterm birth, congenital defects, and perinatal death (Gaillard et al., 2013). A body of birth cohort studies demonstrates that over-nutrition during pregnancy increases the susceptibility to metabolic diseases in offspring later in life (Eriksson et al., 2014; Gaillard et al., 2014; Hussen et al., 2015). Thus, the effects of maternal obesity are particularly important for determining health outcome of the progeny (**Table 1**).

**Table 1:** Maternal dietary factors link epigenetics and microbiome to obesity in the offspring.

Maternal diets or supplementation	Epigenetic mechanisms	Microbiota	Specific gene changes	Clinical effects in offspring
Over nutrition (e.g., high fat diet [HFD])	Induce altered DNA methylation, histone acetylation and histone methylation patterns in offspring epigenome (Aagaard-Tillery et al., 2008; Dudley et al., 2011; Suter et al., 2012; Lee, 2015; Masuyama et al., 2015; Indrio et al., 2017)	Altered gut microbiome profiles in the offspring (Chu et al., 2016b; Zhou and Xiao, 2018)	Adipogenesis-related genes such as *adiponectin, leptin*, and *PPAR-γ* (Aagaard-Tillery et al., 2008; Dudley et al., 2011; Suter et al., 2012; Masuyama et al., 2015)	Increased birth body weight and high risk of development of metabolic disorders and obesity in later life (Parlee and MacDougald, 2014)
Under nutrition	Induce DNA methylation and histone modification changes as well as microRNA profile changes (Fu et al., 2004; Tosh et al., 2010; Slater-Jefferies et al., 2011; Zheng et al., 2017)	Maternal protein-deficient diets modulate gut microbiome profiles in the offspring (Chu et al., 2016b)	Adipogenesis- and metabolism-related *IGF-1, PPAR-α, CPTI C/EBPβ* genes (Fu et al., 2004; Tosh et al., 2010; Slater-Jefferies et al., 2011; Zheng et al., 2017)	Maternal food restriction resulted in fetal intrauterine growth restriction (IUGR) and increased susceptibility to insulin resistance and diabetes in the offspring (Li et al., 2010; Parlee and MacDougald, 2014)
Methyl donor (e.g., folate acid)	Restriction of maternal folate resulted in widespread epigenetic alterations in DNA methylation (Sinclair et al., 2007)	Folate is produced by gut *Bifidobacteria* (Pompei et al., 2007)	Maternal folate deficiency induced hyperacetylation of *PPAR-γ* coactivator, *PGC-1α* (Gueant et al., 2014)	Maternal folate deficiency resulted in increased risk of insulin resistance, elevated blood pressure and obesity-related metabolic disorders in adult offspring (Gueant et al., 2013; Wang et al., 2016)
Soybean (e.g., genistein)	Maternal soybean genistein led to global modification in the fetal epigenome (Dolinoy et al., 2006)	Soybean genistein is bio-converted by gut microbiome and it can also modulate the microbiome profiles (Paul et al., 2015, 2017)	Maternal soybean genistein resulted in hypermethylation of the ectopic *agouti* gene expression (Dolinoy et al., 2006)	Maternal soybean genistein led to reduction of prevalence of obesity in the mouse offspring (Dolinoy et al., 2006)
Cruciferous vegetables (e.g., sulforaphane)	Sulforaphane is a potent histone deacetylase inhibitor and maternal broccoli sprouts lead to global epigenetic changes in mouse offspring (Myzak et al., 2004; Li et al., 2014; Li et al., 2018)	Sulforaphane is bio-converted by gut microbiome and it also modulates the microbiome profiles (Paul et al., 2015)	Tumor-related genes such as *p53, p16, c-Myc*, and *hTERT* (Li and Tollefsbol, 2010; Hardy and Tollefsbol, 2011; Li et al., 2014)	Sulforaphane counteracts HFD-induced body weight and metabolic disorders (Nagata et al., 2017)
Green tea polyphenols (e.g., EGCG)	EGCG is a DNA methyltransferase inhibitor and can induce reactivation of DNA methylation-silenced gene expression (Fang et al., 2003). Green tea polyphenol EGCG inhibits maternal HFD-induced neural tube defects by inhibiting DNA hypermethylation (Zhong et al., 2016).	Green tea polyphenols reversed HFD-induced gut microbial diversity changes by decreasing *Firmicutes* to *Bacteroidetes* ratio (F/B ratio) in C57BL/6J mice (Wang et al., 2018).	Tumor suppressor genes such as *p16, RNRβ, MGMT*, and *hMLH1* (Fang et al., 2003); Neural tube closure essential genes, including *Grhl3, Pax3*, and *Tulp3* (Zhong et al., 2016)	Small amount green tea during pregnancy may provide transplacental protection against carcinogenesis and obesity in the offspring (Castro et al., 2008)
Probiotics	Various metabolites of gut microbiota such as short-chain fatty acid (SCFAs) and butyrate can influence epigenetic pathways (Pompei et al., 2007; Hamer et al., 2008; Berni Canani et al., 2012; Paul et al., 2015; Cortese et al., 2016).	*Lactobacillus* and *Bifidobacterium* (Chu et al., 2016b; Indrio et al., 2017; Zhou and Xiao, 2018)	Probiotic supplementation during pregnancy affects DNA methylation status of certain promoters of obesity and weight gain-related genes both in mothers and their children (Luoto et al., 2010; Wickens et al., 2017; Vähämiko et al., 2018)	Probiotic intake during pregnancy and lactation attenuates maternal HFD-induced detrimental nutritional programming of offspring obesity (Paul et al., 2016)


Moreover, a variety of animal studies have strengthened the importance of maternal nutrition and demonstrated the relationship between maternal over-nutrition and progeny obesity with concrete mechanisms. These studies indicate that dams fed maternal high-fat diet (HFD) usually develop a poor fetal intrauterine developmental environment, and more likely give birth to pups with higher body weight and metabolic disorders including elevated circulating glucose, leptin, triacylglycerols, and cholesterol after weaning compared to pups born to regular chow-fed controls (Samuelsson et al., 2008; Friedman, 2015; Bringhenti et al., 2016). It is suggested that maternal obesity is correlated with impaired insulin signaling pathways, increased pancreatic beta cell mass and altered islet structure (Ford et al., 2009). In the long-term, it increases the risk of diabetes and non-alcoholic fatty pancreas disease in adult offspring.

Indeed, the mechanisms through which the high risk of obesity and metabolic disorders in the offspring induced by maternal HFD may be partially due to the altered epigenome from the obese mother affecting the fetal epigenome during early development. Studies performed in primates have shown that an energy dense HFD can alter fetal chromatin structure leading to an increased recruitment of transcription factors to the target DNA binding sites via changing histone modification patterns (Aagaard-Tillery et al., 2008). It was found that a maternal HFD in primates can modulate fetal type III histone deacetylase, sirtuin 1 (*SIRT1*), which links to regulation of the fetal epigenome and metabolome (Suter et al., 2012). In rodents, Masuyama et al. (2015) showed that maternal HFD can decrease adiponectin but increase leptin gene expression partially due to an increased enrichment of the transcriptional repressor, dimethyl-histone 3 lysine 9 (dimethyl-H3K9), in the promoter of the adiponectin gene and increased enrichment of the transcriptional activator, monomethylated histone 4 lysine 20 (monomethyl-H4K20), in the promoter of the leptin gene, respectively. Maternal HFD can also alter DNA methylation patterns in other important organs involved in metabolism such as liver (Dudley et al., 2011).

However, a single maternal HFD may not be sufficient to produce transgenerational effects on offspring obesity since body weight increases were only found in the first, but not in the second generation of female and male offspring based on Dunn’s study (Dunn and Bale, 2009). This may partially explain the findings that the observed phenotypes and epigenetic changes can be corrected when a normal maternal diet is administered in the subsequent generations, indicating no permanent epigenetic imprinting is inherited from a maternal HFD stress. It also provides evidence that a lower fat diet may help correct energy surfeits-induced epigenetic modification from previous generations and decrease risk of obesity and obesity-associated metabolic dysfunction.

Although food preference will likely maintain the same during gestation and lactation in western countries, most Asian women commonly change their diets to high-fat, high-protein and high-calories diet exclusively during lactation with the belief that this will help stimulate milk production and provide more nutrients to the newborn baby. During this postpartum period, Asian mothers undergo ‘confinement,’ a set of practices including dietary changes and restricted movement to assist them in recovery from pregnancy and childbirth (Dennis et al., 2007). However, this traditional practice may raise a concern since adverse effects may occur. Animal results indicate that consumption of HFD during lactation impaired mammary parenchymal tissue and impeded its ability to synthesize and secrete milk, and impaired thermogenic function of brown adipose tissue resulting in metabolic disorder in the offspring (Liang et al., 2016). In addition, confinement-induced maternal obesity during lactation can increase the leptin surge and induce prolactin resistance, which further deteriorated the metabolic system and increased obesity risk in the offspring (Masuyama and Hiramatsu, 2014; Buonfiglio et al., 2016).

In summary, women are encouraged to maintain normal body mass index (BMI) and healthy/balanced diets during pregnancy and lactation not only for their own health perspective but also for protecting their progenies against obesity and obesity-associated metabolic disorders and chronic disease in their adult life.

### Maternal Under-Nutrition or Nutrition Deficiency

Although food shortages and nutrition deficiency are not a pervasive problem in industrialized nations, it used to be very common in human history. The Dutch famine has set a good example by showing how maternal malnutrition influences later-life obesity risk in progenies. Epidemiological studies have shown that the individuals whose mothers had undergone a significant calorie reduction by over 50% during the Dutch famine displayed increased rates of obesity and metabolic disorders such as glucose intolerance than control counterparts (Lumey et al., 1993). These results suggest that maternal nutrient restriction is a powerful force to induce an opposing effect on the propensity to develop obesity (**Table 1**).

Remarkably, epigenetic mechanisms have been shown to play a major role in the regulation of maternal malnutrition-induced negative impact on relevant gene expressions, resulting in possible long-term consequences in the offspring (Li et al., 2010). Epigenetic mechanisms linking maternal under-nutrition (either global or protein-restriction) to later-life phenotypes have been extensively explored in animal models (Fu et al., 2004; Tosh et al., 2010). Studies in experimental animals such as rodents and sheep have shown that maternal malnutrition can induce intrauterine growth restriction (IUGR) leading to a series of adverse effects including obesity in the offspring. In experimental models of IUGR, uteroplacental insufficiency can induce site-specific changes in histone H3 modifications leading to decreased expression of postnatal insulin-like growth factor-1 (*IGF1*) mRNA in rat liver (Fu et al., 2004). Moreover, IUGR-altered hepatic *IGF-1* mRNA expression was due to histone H3K4 methylation pattern changes that may contribute to early epigenetic reprogramming toward obesity in the rat (Tosh et al., 2010). Maternal low-protein diets bring additional evidence for epigenetic effects of maternal nutrition deficiency on the health of the offspring. For example, a protein-restricted diet during pregnancy can epigenetically affect key adipogenesis genes such as peroxisomal proliferator-activated receptor-α (*PPAR-α*) or energy homeostasis gene, contributing to maternal nutrition deficiency-led cardiomyopathy in the offspring (Slater-Jefferies et al., 2011). In addition, maternal low-protein diets can also regulate microRNA expressions, which are associated with chronic inflammation status and metabolic health in offspring (Zheng et al., 2017). Macroscopically, maternal nutrient-restriction during gestation (either global or low protein) caused a decrease in pancreatic β-cell mass and dysfunction, which may also contribute to the glucose intolerance identified in the adult animals (Dumortier et al., 2007).

Thus an early-life nutritional restriction is considered as an important environmental factor to force a thrifty metabolic phenotype. However, it can create a shift in a hypercorrection direction to the developmental origin of diseases such as obesity.

### Epigenetic Bioactive Diets

Nutrition as one of the most important environmental factors frequently influences gene expression through regulation of epigenetic mechanisms. A number of studies have shown certain bioactive dietary components in our daily diets are able to exert their chemopreventive effects against various human diseases through modulation of epigenetic pathways (Li and Tollefsbol, 2010; Hardy and Tollefsbol, 2011). These particular bioactive diets are referred as “epigenetic diets” including methyl donors (e.g., folate and vitamin B12) enriched diets, green tea polyphenols, soybean isoflavone and broccoli sprouts sulforaphane et al (Hardy and Tollefsbol, 2011). These epigenetic diets can influence the initiation processes of epigenetic hallmarks during early life via maternal exposure leading to long-term effects on human diseases (**Table 1**).

One-carbon metabolism is an important metabolic process for DNA methylation and has profound impact on the development of many human diseases. Folic acid as one of the well-studied methyl donors is crucial for synthesis of S-adenosylmethionine (SAM), the universal methyl donor of DNA methylation and synthesis (Hoffman et al., 1979). The role of folate is especially critical during early embryonic and postnatal development. Folic acid deficiency during gestation has been shown to associate with an increased risk of several birth defects (McKay et al., 2004). In sheep, it was found that restriction of folate and methionine during the periconceptional period resulted in widespread epigenetic alterations in DNA methylation and altered health-related phenotypes such as insulin resistance and elevated blood pressure observed in adult male offspring (Sinclair et al., 2007). In addition, maternal deficiencies in folate and vitamin B12 during gestation and lactation led to further metabolic disorders in key metabolic organs such as increased central fat mass and liver steatosis in the offspring (Gueant et al., 2013). Consistently, a recent prospective birth cohort indicates that maternal folate deficiency can increase obesity-induced child metabolic risk; conversely, sufficient maternal folate concentrations can mitigate the detrimental effects of maternal obesity in the offspring (Wang et al., 2016). The underlying molecular mechanisms may be related to altered metabolism-related key gene expression such as *PGC1-α* through epigenetic regulation due to a decrease in SAM and genome-wide hypomethylation (Gueant et al., 2014).

Recent studies have shown that many epigenetic diets such as green tea, broccoli sprouts, soybean and grapes as well as the bioactive compounds extracted from these diets exert their profound actions on prevention of various human diseases, such as cancer, obesity, and diabetes by reversing aberrant epigenetic profiles (Li and Tollefsbol, 2010; Hardy and Tollefsbol, 2011). These epigenetic diets may also have an impact on developmental processes leading to disease prevention in later life if the dietary exposures occur during the critical developmental stages such as early embryogenesis and/or the early postnatal period (Li et al., 2014; Lee, 2015).

Soybean products and their derived bioactive isoflavones such as genistein have been of particular interest because they are associated with a lower rate of breast cancer in Asian women (Barnes, 1995). In addition to its anti-cancer property, genistein is believed to be a chemopreventive agent against various human diseases including obesity via mechanisms including inhibiting adipogenesis, activating the AMPK pathway and reducing oxidative stress in obese mice (Behloul and Wu, 2013). Several studies have shown that maternal consumption of dietary genistein led to phenotype alterations in the mouse offspring such as reduction of prevalence of obesity due to hypermethylation of the ectopic *agouti* gene expression by modifying the fetal epigenome (Dolinoy et al., 2006). However, a recent study shows that postnatal exposure to soybean genistein increased risk of obesity development in female offspring and the mechanisms may involve activation of adipogenic genes via regulation of DNA methylation (Strakovsky et al., 2014). These results suggest the importance of exposure window of soybean genistein in maternal diets that may lead to different health outcomes in later life.

Sulforaphane, a phytochemical isothiocyanate enriched in cruciferous vegetables such as broccoli sprouts (BSp), cabbage and kale, is believed to reduce the risk of obesity. A recent study demonstrates that mice fed with sulforaphane can counteract HFD-induced body weight gains by a 15% reduction, and a 20% visceral fat reduction, as well as reduction of hepatic steatosis and blood glucose level than that of the mice fed without sulforaphane (Nagata et al., 2017). As an important histone deacetylase (HDAC) inhibitor (Myzak et al., 2004), sulforaphane has been studied for its effects in regulation of epigenetic mechanisms and potential roles on transplacental or transgenerational prevention effects on disease development (Li et al., 2014). Epidemiological studies indicate that Asian populations who consume more cruciferous vegetables at earlier ages than Caucasians have less incidence of breast cancer, suggesting beneficial effects of maternal consumption of cruciferous vegetables during pregnancy (Lee et al., 1991). Our recent studies also reveal that maternal administration of sulforaphane-enriched broccoli sprouts diet led to a temporal effect on prevention of breast cancer through regulation of epigenetic pathways in mouse offspring (Li et al., 2018). However, direct evidence linking maternal broccoli sprouts to obesity prevention in the offspring is still under investigation.

Bioactive compounds such as the green tea polyphenol, epigallocatechin-3-gallate (EGCG), is a potent DNA methyltransferase (DNMT) inhibitor. It has been shown to reverse methylation-induced silencing and change the expression of various tumor suppressor genes that contributes to its chemopreventive effects (Fang et al., 2003; Li and Tollefsbol, 2010). Caffeine-enriched beverages such as green tea and coffee are commonly consumed by pregnant mothers in European countries. Although consumption of catechol-containing polyphenols has shown adverse effects on fetal development, a small amount of green tea (<2 cups per day) is considered safe during pregnancy and may provide transplacental protection against carcinogenesis and obesity in the offspring (Castro et al., 2008). Green tea polyphenol EGCG can also inhibit maternal HFD-induced neural tube defects by inhibiting DNA hypermethylation status and the restoration of neural tube closure essential gene expression, including *Grhl3, Pax3*, and *Tulp3*, indicating its potent *in utero* protection property (Zhong et al., 2016).

These findings demonstrate that appropriate maternal dietary nutritional exposure during a crucial time can alter epigenetic activities-associated reprogramming processes and disease phenotypes, which may lead to different susceptibilities to diseases in late life such as obesity.

## Maternal Diet, Epigenetics and Gut Microbiome in Development of Later-Life Obesity

### Early-Life Gut Microbiome Establishment

Although the gastrointestinal system functions to absorb and excrete food and nutrition we consume everyday, it is also considered to be the most important habitation for the indigenous intestinal microbiota. Recent studies have indicted that the establishment of the gut microbiome through microbial transmission from mother to offspring during early-life may have a major impact on human pathogenesis in later life (Tamburini et al., 2016).

In contrast to the traditional “sterile womb” concept, more compelling evidence shows that the microbiota exists in the placenta, amniotic fluid, umbilical cord blood, and meconium in healthy gestations supporting the “*in utero* colonization” hypothesis. It indicates that the offspring microbiota is transmitted from the mothers and inhabit the *in utero* environment prior to birth (Funkhouser and Bordenstein, 2013; Perez-Muñoz et al., 2017). However, the precise route of transmission to date remains unclear.

In addition to intrauterine “infection,” the postnatal stage during lactation is also important for establishment of gut microbiota composition and can be influenced by several factors such as contact types with the mother, maternal diets and breastfeeding/formula (Chu et al., 2016b). It is widely accepted that the offspring microbiota is essential for the establishment and development of a healthy immune and metabolic function and further health in the offspring (Neu, 2015). Hence, maternal microbiota could serve as an original resource for early establishment of gut microbiota in the offspring. Maternal nutritional exposures during pregnancy and lactation could impact microbial transmission during this period, leading to modified microbial composition and long-term consequences toward more pathogenic profiles on health or disease in the offspring (**Figure 1**).

### Interaction of Maternal Diet and Gut Microbiome Links to Later-Life Obesity

As aforementioned, maternal diet and early-life nutrition status acts as important environmental factors that can influence the risk of developing various human diseases in later life including obesity and its associated metabolic disorders in adulthood. Importantly, the gut microbiome plays a significant role in many physiological functions including facilitating digestion of nutrients including fats and carbohydrates. Thus, it is closely associated with maternal and offspring obesity (Chu et al., 2016b; Zhou and Xiao, 2018).

Substantial human and animal studies have shown the altered composition and diversity of the gut microbiota in obese populations (Kvit and Kharchenko, 2017). However, the mechanisms by which the host diets impact the composition of the intestinal microbiome remain to be fully elucidated. At the phylum level, *Firmicutes* and *Bacteroidetes* are the dominant bacteria that compose more than 90% of human gut microbiota (Human Microbiome Project Consortium, 2012). Notably, recent studies indicate that the *Firmicutes* to *Bacteroidetes* ratio (F/B ratio) is correlated with obesity (Ley et al., 2005). Specifically, obese individuals tend to harbor an increased F/B ratio (**Table 2**). Studies have shown that transplantation of gut microbiome from diet-induced obese mice to lean germ-free recipients resulted in a significant increased body fat deposition suggesting an important role for the intestinal microbiome in development of obesity (Turnbaugh et al., 2008). Strikingly, studies in humans indicate that patients with metabolic syndrome who received an infusion of microbiota from lean donors showed a significant increase in insulin sensitivity partially due to increased population of butyrate-producing intestinal microbiota (Vrieze et al., 2012). Therefore, transplantation of specific gut microbiota could serve as a potential therapeutic strategy for treatment of certain metabolic disorders such as obesity in humans.

**Table 2:** Specific profiles of gut microbiota in regulation of maternal diets-induced obesity in the offspring.

Microbial profiles	Maternal diet effects	Impacts on metabolism	Epigenetic mechanisms
*Firmicutes/Bacteroidetes*	An increased ratio of intestinal *Firmicutes* to *Bacteroidetes* (F/B ratio) is associated with obesity (Ley et al., 2005). Reversed F/B ratio in HFD-induced obesity by green tea polyphenols (Wang et al., 2018)	Green tea polyphenols alter gut microbiota diversity and lipid metabolism (Wang et al., 2018).	An improved gut microbial balance by diets may result in epigenetic changes in metabolic genes leading to prevention of metabolic syndrome (Paul et al., 2015; Wang et al., 2018).
*Proteobacteria*	Decrease in preterm placentas with excess gestational weight gain (GWG) (Antony et al., 2015); Depletion in offspring gut with maternal HFD (Ma et al., 2014)	Decreased metabolisms in folate biosynthesis and butyrate in preterm placentas with excess GWG (Antony et al., 2015)	Butyrate influences histone modification and folate is a methyl-donor that can influence epigenetic processes (Hoffman et al., 1979; Berni Canani et al., 2012; Paul et al., 2015). Decreased butyrate production and folate bioavailability may result in dysregulation of epigenetic profiles in offspring
*Bifidobacterium*	Depletion in offspring with a maternal HFD (Paul et al., 2018); Gestation/lactation reintroduction leads to ameliorate maternal HFD-induced detrimental nutritional programming of offspring obesity (Luoto et al., 2010; Vähämiko et al., 2018); Increase by soybean intake (Paul et al., 2017)	Decreased production of butyrate and folate bioavailability	1. Butyrate can epigenetically regulate obesity-related gene expressions such as *PPAR*γ and *interferon-γ* (Berni Canani et al., 2012). 2. Butyrate can affect DNA methylation processes by regulating methyl-donor availability through regulation of folate production (Pompei et al., 2007); 3. Supplementation of *Bifidobacterium* during pregnancy affects DNA methylation status of certain promoters of obesity and weight gain-related genes both in mothers and their children (Luoto et al., 2010; Vähämiko et al., 2018)
*Lactobacillus*	Depletion in offspring with a maternal HFD (Buffington et al., 2016); Gestation/lactation reintroduction leads to ameliorate maternal HFD-induced detrimental nutritional programming of offspring obesity (Luoto et al., 2010; Wickens et al., 2017; Vähämiko et al., 2018)	Decreased production of butyrate, a histone deacetylase (HDAC)	1. Butyrate epigenetically upregulates gene expressions of *PPAR*γ and decreasing *interferon-γ* production (Berni Canani et al., 2012). 2. Supplementation of *Lactobacillus* during pregnancy affects DNA methylation status of certain promoters of obesity and weight gain-related genes both in mothers and their children (Luoto et al., 2010; Wickens et al., 2017; Vähämiko et al., 2018)
*Campylobacter*	Decrease by gestational/postnatal HFD in offspring (Ma et al., 2014)	The presence of *Campylobacter* in HFD/CDT offspring positively correlates with pathways in amino acid metabolism, carbohydrate metabolism, and lipid metabolism (Ma et al., 2014)	n/a
*Clostridium*	Decrease in obese dams from pre-pregnancy to lactation (Paul et al., 2018)	Dysregulated one-carbon and mammary gland metabolism	One-carbon cycle will generate the universal methyl donor S-adenosylmethionine (SAM), which is vital for DNA methylation process (Hoffman et al., 1979)


Because of the fact that maternal HFD could program a significantly increased predisposition to obesity and metabolic disorders in the offspring, recent studies have highlighted the importance of maternal obesity, featured as excess gestational weight gain (GWS), which is associated with alterations in the placental microbiome contributing to obesity in the offspring (**Table 2**; Antony et al., 2015). In a recent mouse study, maternal HFD detrimentally altered gut microbiome profiles in the offspring, which contributed to development of obesity, steatohepatitis and its progressive diseases in the offspring (Paul et al., 2016, 2018; Wankhade et al., 2017). Intriguingly, studies in non-human primates have shown that maternal HFD led to persistently reduced abundance of *Campylobacter* species in the offspring gut compared to the control diet group even though the primates were weaned and switched to control diet for 6 months (**Table 2**; Ma et al., 2014). This study provides early evidence that maternal diet can permanently shape the commensal microbiome profiles in the offspring gut. This original seeding of the microbiome from maternal transmission may play profound roles in influencing development of human diseases in later life. Similarly, human studies also highlight this conclusion indicating that maternal obesity and over-nutrition can alter the gut microbiome in the offspring, which partially explains the consequence of an increased risk of obesity in children with obese mothers (Chu et al., 2016a).

Gut microbiota also participates in metabolism of many epigenetic diets in our body system (**Table 1**). For example, bacteria such as *Bifidobacterium* strains can facilitate producing folate and butyrate in the human intestine (Picard et al., 2005). Broccoli sulforaphane can be converted to biologically active compounds by β-thioglucosidases produced by the gut microbiome (Paul et al., 2015). Moreover, ingested soybean genistein can be absorbed and biotransformed by intestinal microflora to become biologically active through bacterial produced β-glucosidases (Paul et al., 2015). These bioactive dietary components converted by intestinal microbiome can further reach the circulation system and exert their biological function.

It is believed that the diet can also determine the dominant bacterial strains in the gastrointestinal tract. Our recent studies show that soybean genistein modulated the microbiome profiles in breast cancer patients’ fecal sample-humanized mice which may have contributed to its effects on inhibiting breast tumor development (Paul et al., 2017). In addition to genistein, equol, as one of the important metabolites of soybean isoflavone, has received considerable attention due to its greater affinity for estrogen receptors, unique antiandrogenic properties and superior antioxidant activity (Yuan et al., 2007). However, only approximately one third of the human population is able to metabolize daidzein to equol and this difference is presumably due to different composition of the intestinal microbiome, which may play an important role in metabolizing soy isoflavones. Because both genistein and equol are able to transfer from mother to fetus through the placenta, on the fetal side, soybean isoflavones will be further metabolized and excreted through microbiota in amniotic fluid (Todaka et al., 2005). Thus it is possible that the offspring can “inherit” special microbial species from the mother *in utero* to help them efficiently metabolize soybean isoflavone that benefit their future health. However, the transmission route, the metabolism mechanism in the fetus and the effects of fetal exposure to bioactive diets need be further studied. Other bioactive epigenetic diets such as green tea polyphenols have been reported to improve HFD-induced metabolic disorders partially through regulation of gut microbial diversity (Wang et al., 2018), although the effects of maternal green tea polyphenols on the fetus microbiota remains unclear.

The impact of maternal nutrition on the offspring guts microbiome and the associated health outcomes can extend beyond pregnancy into the postnatal period through breastfeeding. Maternal HFD during lactation can significantly influence fat content and alter bacterial composition in human milk, leading to microbiota composition shifts in the offspring gut (Robinson and Caplan, 2015). Cross-fostering studies in mice born to the normal diet-fed mother but breastfed by the HFD mother have highlighted this conclusion indicating the importance of maternal diet during lactation to offspring health (Du et al., 2015).

In addition to maternal diet, the delivery method also contributes to early microbiome establishment. Babies delivered by cesarean section (C-section) acquire a microbiota that differs from that of vaginally delivered infants (Tamburini et al., 2016). Many studies have showed that C-section delivery is associated with increased risk of obesity in later life, which may be due to a delayed acquisition of beneficial bacterial colonization such as *Bifidobacteria* in the newborn guts (Grönlund et al., 1999; Goldani et al., 2011). A recent study showed that the microbiota can be partially restored if the C-section-born infants were exposed to maternal vaginal fluids at birth (Dominguez-Bello et al., 2016). It raises a speculation that the beneficial or adverse maternal dietary effects on babies’ health outcome may be enhanced or blocked by microbiota transfer at birth. However, further studies are needed to demonstrate the feasibility of this provocative approach.

### Gut Microbiome and Epigenetic Mechanisms

It has been widely accepted that the gut microbiome can induce epigenetic changes in the host and the interaction between the commensal microbiota and the epigenome of the host contribute to disease etiology (Cortese et al., 2016; Wankhade et al., 2017). Although the underlying mechanisms by which the gut microbiome influences epigenetic mechanisms still need to be elucidated, current evidence supports a significant correlation between gut microbiota composition and epigenetic changes in specific genes relevant to a series of human diseases (Paul et al., 2015). The microbiota-produced bioactive metabolites from diets such as folate, butyrate, biotin, acetate, and acetyl-CoA may also participate in regulation of epigenetic processes. For example, butyrate, as a short-chain fatty acid (SCFA), is one of the most important metabolites to maintain colon homeostasis, which is produced by beneficial colonic bacteria such as *Bifidobacterium, Anaerostipes, Eubacterium*, and *Roseburia* species (Hamer et al., 2008). Most importantly, butyrate is a histone deacetylase (HDAC) that can influence histone modification processes. Studies have shown that butyrate production can influence metabolism by epigenetically regulating obesity-related gene expressions such as *PPAR*γ and interferon-γ (Berni Canani et al., 2012). In addition, butyrate can affect DNA methylation processes by regulating methyl-donor availability through regulation of folate production (Pompei et al., 2007). Thus, decreased butyrate production caused by imbalance of gut microbiome may result in dysregulation of epigenetic profiles in the offspring (**Table 2**). This suggests a key connection of diets, especially diets consumed during critical time intervals such as prenatal/maternal/early postnatal periods to establishment of early-life mocribiota habitation, epigenetic profiles and further health outcomes.

The early-life maternal diets, microbiome and epigenome may interact with each other and establish a complicated crosstalk mechanism that contributes to initiation of developmental plasticity in response to the environmental factors (**Figure 1**). The optimal/detrimental environmental factors such as maternal beneficial diets/HFD may lead to the establishment of the diverse microbiomic and epigenomic profiles leading to different health outcomes such as prevention effects or subsequent susceptibility to disease or disorders including obesity later in life.

## Preventive Implications of Early Obesity Interventions

Evidence on early-life obesity programming provides promising prevention approaches that center on intervention via maternal diets, gut microbiota and epigenetic mechanisms. As mentioned previously, maternal diets containing bioactive components that can influence epigenetic regulations have shown beneficial effects on prevention of many human diseases including obesity (Li and Tollefsbol, 2010; Hardy and Tollefsbol, 2011). Consumption of these epigenetic diets including, but not limited to, folate-enriched leafy vegetables, soybean, broccoli sprouts and green tea polyphenols, may be recommended for optimal maternal diets due to their beneficial effects on prevention of later-life obesity and metabolic disorders in experimental animals and epidemiological studies (Li et al., 2014; Parlee and MacDougald, 2014; Lee, 2015). However, particular mechanisms for precise dose and exposure windows should also be addressed and studied in the future.

An altered gut microbiome in early life owing to maternal diets provides additional mechanisms for obesity etiology. Further alternative approaches for early prevention of obesity are achievable by introduction of optimized panels of microbiota such as probiotics to correct potential adverse effects during early life. Studies have shown that prenatal probiotic supplements such as *Lactobacillus* and *Bifidobacterium* during pregnancy restrain gestational weight gain and blood glucose level, and improve insulin sensitivity metabolism, which may have a beneficial impact on the metabolic system against obesity in offspring (Luoto et al., 2010; Wickens et al., 2017; **Table 2**). In a recent animal study, researchers found that probiotic intake during pregnancy and lactation attenuated maternal HFD-induced detrimental nutritional programming of offspring obesity associated with maternal obesity, indicating that altered maternal gut microbiota through additional microbiota manipulations could be a useful strategy to improve maternal and offspring metabolic outcome with a long-standing impact (Paul et al., 2016). A pilot human study indicates that probiotic supplementation during pregnancy may affect the DNA methylation status of certain promoters of obesity and weight gain-related genes both in mothers and their children (Vähämiko et al., 2018). This provides strong evidence demonstrating potential crosslink mechanisms between gut microbiota composition, epigenetic regulation and obesity incidence in humans. Thus, optimized maternal diets and subsequent gut microbiota modification might influence the developmental programming of obesity, which may provide compelling implications for future nutrient and dietetic instruction for the clinical OB/GYN practices.

## Conclusion

It is well-established that the maternal diet may have a long-term impact on offspring health outcome in adulthood. In light of this factor, nutrient supply during pregnancy and lactation is especially important to prevent adverse fetal programming and susceptibility to later-life diseases, among which, obesity is of particular importance. Obesity has strong genetic/epigenetic predisposing tendencies that are frequently affected by earl-life maternal nutritional status. Due to a prevalence of obesity in pregnant women, early-life maternal dietary intervention has tremendous social/economic impact on prevention of global obesity prevalence in our society. The particular crosstalk between the maternal diets, epigenetic reprogramming and gut microbiome provides an excellent intervention strategy via modulation of these mechanisms in order to interfering with early obesity programming. Maternal diet/calorie control, epigenetic diets and probiotic supplements could shed considerable light on future obesity intervention. Additionally, future studies will help to explore precise mechanisms and facilitate more accurate intervention strategies including effective/optimal exposure doses, temporal schedules, routes of administration, combinatorial approaches to meet individual need leading to prevention of obesity seeding in early life.

## Author Contributions

YL conceived and drafted the manuscript.

## Conflict of Interest Statement

The author declares that the research was conducted in the absence of any commercial or financial relationships that could be construed as a potential conflict of interest.
